# Comparison of two invasive non-surgical treatment options for uterine myomas: uterine artery embolization and magnetic resonance guided high intensity focused ultrasound—systematic review

**DOI:** 10.1186/s12905-022-01627-y

**Published:** 2022-03-03

**Authors:** Madina Yerezhepbayeva, Milan Terzic, Gulzhanat Aimagambetova, Byron Crape

**Affiliations:** 1 Department of Medicine, School of Medicine, Nazarbayev University, Kabanbay Batyr Avenue 53, Nur-Sultan, Kazakhstan; 2 Clinical Academic Department of Women’s Health, Corporate Fund “University Medical Center”, Turan Ave. 32, Nur-Sultan, Kazakhstan; 3 Department of Obstetrics, Gynecology and Reproductive Sciences, School of Medicine, University of Pittsburgh, 300 Halket Street, Pittsburgh, PA 15213 USA; 4 Department of Biomedical Sciences, School of Medicine, Nazarbayev University, Kabanbay Batyr Avenue 53, Nur-Sultan, Kazakhstan

**Keywords:** Magnetic resonance guided high intensity focused ultrasound, Uterine artery embolization, Uterine leiomyoma, Uterine fibroid

## Abstract

**Background:**

Uterine Artery Embolization (UAE) and Magnetic Resonance guided High Intensity Focused Ultrasound (MRgHIFU) are two noninvasive treatments for uterine leiomyoma.

**Methods:**

This systematic review, following PRISMA guidelines, analyzed the effectiveness of two treatments by comparing percent fibroid volume shrinkage immediately after the procedure and after 3, 6, 12 and 24 months of follow-up and also assessed and compared common complications following treatment. The search utilized Science Direct, PubMed, MEDLINE, Google Scholar and BioMed Central databases, selecting manuscripts published during the period 2000 and 2020. Studies with premenopausal patients with previous treatments for uterine leiomyoma and/or with other pelvic diseases were excluded.

**Results:**

Twenty-nine papers satisfied inclusion and exclusion criteria. Results were pooled and stratified by treatment and follow-up time. Weighted fibroid volume percent shrinkage after UAE was statistically significantly greater than MRgHIFU at 6, 12, and 24 months follow-up times. However, UAE had statistically significantly more complications, such as pain, nausea and vomiting. However, this study cannot conclude that UAE is more effective than MRgHIFU due to confounding factors.

## Introduction

Uterine leiomyoma or uterine fibroids are the most prevalent benign smooth-muscle tumors of the uterus. They are present in approximately 60% of women at reproductive age [1]. However, the real prevalence is likely substantially higher, given that some women do not present symptoms of uterine leiomyomas and thus go undiagnosed. Symptomatic leiomyomas can adversely impact women’s physical, social, and psychological functioning, as well as reduce income and work effectiveness [2]. Symptoms and signs of uterine leiomyoma depend on the type, number, size and secondary changes within the fibroid nodules. Patients with uterine leiomyoma might present with heavy menstrual bleeding, pain, significant intermittent uterine bleeding, iron-deficient anemia, pelvic pain, bowel dysfunction, urinary and pressure symptoms [3]. Uterine leiomyoma is also causally associated with 1–3% of the infertility rate and 8% of miscarriages [4]. Women with uterine leiomyoma have three times higher risk of miscarriage than women without fibroids. An estimated 5–10% of infertile women have uterine fibroids that have contributed to anatomic distortion of the uterine cavity and abnormal endometrial receptivity [5]. Additionally, uterine leiomyoma might cause complications during pregnancy, such as preterm delivery (< 37 weeks), abnormal fetal position, abnormal placentation, placental abruption, postpartum infections and postpartum bleeding [4]. Because of symptoms and complications, the presence of uterine fibroids is the primary indication for conducting a hysterectomy worldwide [6]. Thus, alternative non-invasive or minimally invasive treatments are requisite for avoiding more invasive procedures, while still effectively protecting fertility relieving clinical symptoms for women with uterine leiomyoma.

There are medical, surgical, and minimally invasive treatments for uterine leiomyoma. Asymptomatic patients might not require treatment [2]. The aim of uterine leiomyoma management is to relieve symptoms, avoid or minimize invasiveness, promote rapid recovery following treatment and preserve fertility, if necessary and dependent on the patients’ decisions. Uterine Artery Embolization (UAE) and Magnetic Resonance Guided by High Intensity Focused Ultrasound (MRgHIFU) treatments are minimally invasive procedures that reduce fibroid volume while avoiding the higher risk of uterus damage associated with more invasive procedures. Among the currently available the conservative interventional management options, UAE has the longest history and has been shown to be effective in properly selected patients [3]. Newer focused energy delivery methods are promising but need more investigation on the long-term outcomes [3]. The aim of this systematic review is to analyze and compare fibroid shrinkage following UAE and MRgHIFU treatments and to identify and compare common complications associated with these two procedures. A systematic review was conducted by Taheri et al. [7] assessing fibroid volume changes following UAE and radiofrequency ablation. However, the Taheri systematic review did not take into consideration of either the other treatments and pelvic diseases (except adenomyosis) that patients were experiencing, or the patients’ menopausal or perimenopausal status. Our systematic review evaluates and compares fibroid shrinkage following UAE and MRgHIFU treatments for premenopausal women without the presence of other treatments and other gynecological diseases to avoid confounding biases, and also evaluates and compares common complications for the two procedures.

## Materials and methods

Our systematic review followed PRISMA guidelines utilizing, with searches on Science Direct, PubMed, MEDLINE, Google Scholar and BioMed Central databases to identify published journal articles on related the years 2000–2020. All original research articles, including prospective and historical cohort studies case–control studies, case reports and case series were reviewed. There were no randomized clinical trials. Each individual paper was reviewed by two out of three of the authors that conducted reviews, with any disagreements between these authors being resolved by consultation.

Published manuscripts were considered for inclusion into the systematic review if patients were women with symptomatic uterine leiomyoma who received either UAE or MRgHIFU treatments. Extracted data from the selected studies that met inclusion and exclusion criteria for the systematic review included authors, year of publication, follow-up duration, study design, interventions, participant population, characteristics of patients, size of fibroids, uterine fibroids’ shrinkage after treatment and postoperative outcomes. Outcomes of interest included fibroid volume before and after treatments, mean fibroid volume percent change, and complications following the procedures. Key phrases, including “magnetic resonance guided high intensity focused ultrasound”, “uterine artery embolization” and “uterine fibroids”, and “leiomyoma” or “uterine myomas” were utilized in title searches to identify related research publications.

Our exclusion criteria those studies where patients who have undergone or were undergoing treatments other than UAE or MRgHIFU, patients with other gynecological pathologies that were not uterine leiomyoma, redundant publications with the same data, studies including postmenopausal women, and research reporting fibroid volume changes without providing mean values. Research papers were also excluded if they were published before 2000. Only studies published in English were included. Research publications that met exclusion and inclusion criteria were eligible for inclusion into the systematic review.

Mean fibroid volume percent changes were calculated from baseline, before the procedure, to five different endpoints, of five different follow-up periods including immediately following the procedure, and after 3, 6, 12 and 24 months. A weighted mean was calculated for each follow-up period. The percentage of reported number of occurrences of each complication was calculated for all selected papers for the systematic review.

Data on selected papers were downloaded using the reference manager Mendeley, entered into Microsoft Excel and transferred for analyzed into statistical software STATA version 16.1 [8]. Fibroid volume percent change was compared utilizing a two-tailed *t*-test, with values of *p* ≤ 0.05 being considered statistically significant. The Chi square test was performed to examine statistical significance of the counts differences of complications following the procedures.

To reduce selection bias, full texts of potentially eligible articles were retrieved and independently assessed for eligibility by two reviewers out of three that conducted reviews. Any disagreement between two reviewers over the eligibility of a particular article was resolved through discussion with the third reviewer. Data extracted from selected articles included study design, population characteristics, treatment and outcomes.

Give that this was a systematic review of existing published journal articles and all data utilized was extracted from these studies, the current manuscript was exempt from human subject review, with no consent requirements. The study was conducted without funding.

## Results

**Fig. 1:**
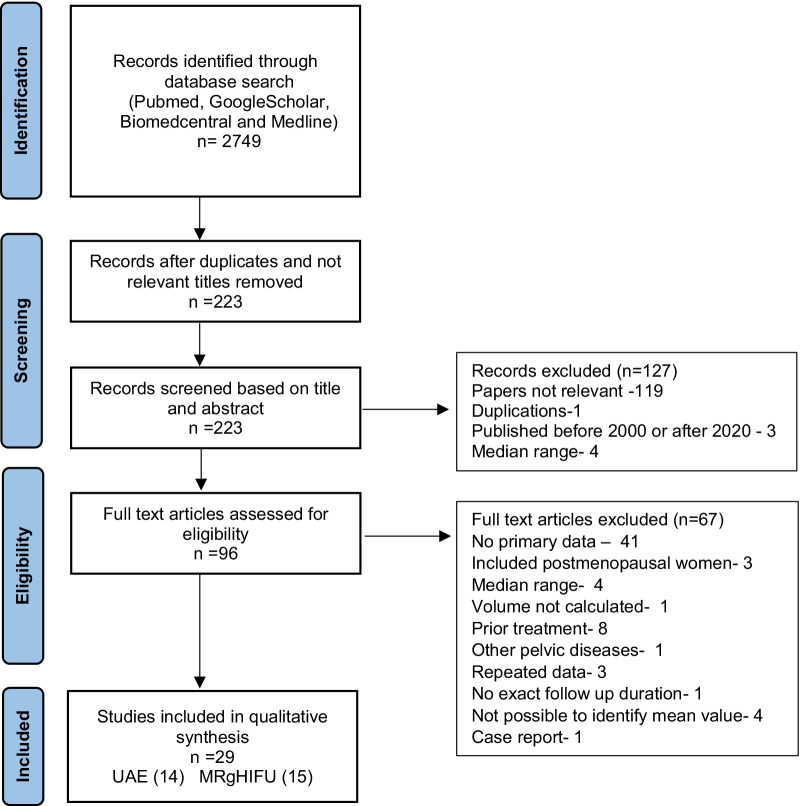
Flowchart diagram to select eligible papers

A total of 2749 papers were identified from the initial search. Out of this number, 29 papers were found eligible for inclusion in qualitative and quantitative analyses, (explained in detail in Fig. 1). Out of the 29 eligible papers, none compared UAE treatment outcomes with that of MRgHIFU. Fourteen case series papers reported UAE treatment outcomes, while 15 reported MRgHIFU treatment outcomes. The sum number of patients who were treated with UAE or with MRgHIFU treatments in the eligible studies was 1383 and 835, respectively. (Table 1).

**Table 1:** Synthesis of data about general features and eligibility criteria of study population treated with UAE

#	References	Study design	Mean age	Number of patients	Number of FB	Inclusion criteria	Exclusion criteria	Mean volume of treated FB	FB shrinkage (%)	Follow up period (months)
1	Burn et al. [9]	PRO	39	18	32	Age 18–53, a	–	340 cm^3^	59	6
2	Roth et al. [10]	PRO	NM	79	NM	–	–	244.5 cm^3^	40	3
3	Klein et al. [11]	PRO	46	35	NM	a, b	–	209 ml	49	6
4	Zupi et al. [12]	PRO	40	26	NM	j	i, m, t	276.8 ± 241.2 ml	55 ± 16.9	6
5	Spies et al. [13]	PRO	43	100	NM	Age 30–55, a, j	d, f, k, n	148.7 ± 153.9 cm^3^	50.06	3
6	Harman et al. [14]	PRO	44	20	28	–	–	123 cm^3^	44.6	6 months
7	Pisco et al. [15]	RETR	41	234	NM	j, l	n	110.5 cm^3^	60.7	6
8	Naguib et al. [16]	RETR	48	28	84	a, b, j	d, c, g (10 cm), n, o, p	51.6 cm^3^	52.62	3
									73.27	12
9	Stampfl et al. [17]	PRO	42	121	NM	Age > 30, b (2 years), j	c, d, i, g, n, o, p, p, u	137.2 ± 245.1 ml	52.4	3
									78.3	6
									91.2	12
10	Bilhim et al. [18]	PRO	39	160	NM	a	–	201.5 cm^3^	53.1	3
									52.95	6
11	Redecha et al. [19]	PRO	NM	98	NM	Age > 18, j	h	NM	68.18	24
12	Song et al. [20]	PRO	43	60	NM	a, j	d, e, i, n, p	224.69 ml	54.05	3
13	Yoon et al. [21]	RETR	42	67	NM	a	d, i, n	143.5 ± 135.4 cm^3^	42 ± 23.1	3
14	Ukybassova et al. [22]	PRO	43	337	NM	Age > 18, j, l	c, d, f, n, o, p, q, r, s	51.53 ± 65.53 mm^3^	9.95	3
									32.18	6
									51.7	12

NM, not mentioned; FB, fibroid; PRO, prospective case series; RETR, retrospective case series

a- fibroid related disease, b- no desire for further pregnancies; c- pedunculated fibroids with more than 50% attachment; d- pregnancy; e- contraindications to MRI and Gadolinium use; f- major medical disease; g- fibroid diameter > 15 cm, h- postmenopause; i- other pelvic diseases; j- premenopause; k-lactating; l- presence of only intramural fibroids, m- subserosal fibroid, n- pelvic inflammatory disease, o- abnormal coagulation status, p- malignancy, q- abnormal endometrial biopsy results, r- abnormal PAP test, s- severe anemia, t- irregular margins and with a sonographic pattern of diffuse fibrosis, u- allergy to contrast material

The overall weighted mean age of patients treated with UAE was not possible to calculate, because not all articles reported patients’ mean age. The weighted mean age of patients who received MRgHIFU treatment was 43 (Table 2).

**Table 2:** Synthesis of data about general features and eligibility criteria of study population treated with MRgHIFU

#	References	Study design	Mean age	Number of patients	Number of FB	Inclusion criteria	Exclusion criteria	Mean volume of treated FB	Fibroid shrinkage (%)	Follow up period (months)
1	Hindley et al. [23]	PRO	45	109	NM	Age > 18, b	f, g, h, i, l, m, n	346 ± 245 cm^3^ (single) 294 ± 188 cm^3^ (multiple)	13.5	6
2	Mikami et al. [24]	RETR	44	48	NM	a, b	e, q	NM	23	6
3	Morita et al. [25]	PRO	43	48	55	Age > 18, b, o	d, i, n, m, s	NM	33 ± 19	6
4	Rabinovici et al. [26]	PRO	46	35	41	a, l	k (20 w), j (10 cm), g, h	216 ± 223 ml	15 ± 27	6
5	Lánä́rd et al. [27]	RETR	45	66	NM	Age > 18, a, b, o	f, g, h, i, l, m, n	255.5 ± 201.7 cm^3^	12.6 ± 16.9	6
									9.3 ± 24.8	12
6	Zhang et al. [28]	PRO	39	21	23	Age > 18, a, e, o	j (10 cm), g, h, u	77.3 ± 66.6 cm^3^	31.4 ± 29.3	3
7	Funaki et al. [29]	PRO	40	91	141	–	f, g, h, i, n, p, s, t	NM	33.1	6
									38	12
									38.2	24
8	Kim et al. [30]	PRO	46	40	51	Age > 18, a, o	c, e, f, g, h, i, k	336 ± 40.8 cm^3^	18.7	6
									25.8	12
									27.6	24
9	Ruhnke et al. [31]	PRO	47 ± 4	18	27	Age 18–59, a, o	c, f, g, h, i, q, w	125 ± 140 ml	45 ± 21	6
10	Thiburce et al. [32]	RETR	44	36	NM	Age > 18, o	f, j, n, r, q	255 (190–319) cm^3^	27	6
11	Tung et al. [33]	PRO	42	40	NM	–	j (10 cm), f, n	258.1 ± 223.8 cm^3^	31.7	6
12	Xu [34]	PRO	42	43	51	Age 18–55, a, b, o	j (12 cm), h, q	NM	33.51	3
									44.52	6
13	Chen et al. [35]	PRO	45	107	130	Age > 18, o, m < 140 kg, l	n, g, l, h	NM	41.6 ± 22.70	3
									50.2 ± 20.40	6
14	Jacoby et al. [36]	PRO	44	13	NM	Age > 18, a, o, b	g, l, h, u, v, j (10 cm),	217 ± 139	18	6
15	Keserci et al. [37]	PRO	40	120	339	Age > 18, a, o	n, g, s, u, h	197.3 ± 155.7 ml	38 ± 26	6

NM, not mentioned, FB, fibroid; PRO, prospective case series; RETR, retrospective case series

a- fibroid related disease; b- no desire for further pregnancies; c- conceive after MRgFUS; d- 3 months treatment GnRH analogue; e- bowel lies anterior to the uterus; f- abdominal scar (locate in the path of the ultrasound beam); g- pregnancy; h- contraindications to MRI and Gadolinium use; i- major medical disease; j- fibroid diameter > 15 cm, k- Uterus larger than 24 gestational weeks, l- hematocrit less than 25%; m- postmenopause; n- other pelvic diseases; o- premenopause; p-lactating; q- degeneration or calcification of fibroids; r- submucosal fibroids, s- pelvic inflammatory disease, t- abnormal coagulation status, u- malignancy, v- abnormal endometrial biopsy results, w- abnormal PAP test

The number of uterine fibroids was not reported in many studies. All papers only included patients older than 18 years of age. The most common reasons for exclusion of patients in both UAE and MRgHIFU studies included pregnancy and restriction size of leiomyoma (10 or 12 cm) or uterine volume measured gestational week (20 week of gestation). Absolute fibroid volume reduction measures were reported in all papers, but only two UAE treatment studies and five MRgHIFU studies reported percentage reductions, which always provided standard deviation or range of values. Some studies included only included measures for the volume of the dominant myoma.

Fibroid shrinkage percentages were stratified by 3, 6, 12 and 24 months follow-up times after treatment (Table 3).

**Table 3:** Pooled data of fibroid volume reductions after UAE and MRgHIFU

Variables	Follow up periods											
	3 months			6 months			12 months			24 months		
	UAE	MRgHIFU	*P* value	UAE	MRgHIFU	*P* value	UAE	MRgHIFU	*P* value	UAE	MRgHIFU	*P* value
Weighted mean fibroid volume reduction ± SD, %	35.59 ± 19.41	38.31 ± 4.29	0.068	50.57 ± 15.70	30.06 ± 12.76	0.0001	62.78 ± 17.10	25.91 ± 12.64	0.0001	68.18	34.96 ± 4.88	0.0001
Minimum	9.95	32.4		32.18	12.6		51.7	9.3		68.18	27.6	
Maximum	54.05	41.6		78.3	50.2		91.2	38		68.18	38.2	
Number of papers	8	3		8	14		3	3		1	2	
Number of patients	952	171		951	810		486	197		98	131	
Number of fibroids	NPC	204		NPC	NPC		NPC	NPC		NPC	192	

NPC, not possible to calculate

Eight UAE and three MRgHIFU treatment papers reported fibroid reductions after 3 months follow-up. The weighted mean difference in fibroid volume reductions between the two treatments after 3 months was not statistically significant (*p* = 0.068). The minimum and maximum percent fibroid volume shrinkage immediately following treatment ranged from 9.95 to 54.04% for UAE and 31.4–41.6% for MRgHIFU.

Twenty-two papers reported fibroid volume shrinkage after 6 months follow-up. These papers included eight papers following UAE treatment and 14 papers following MRgHIFU treatment. At the 6 month follow-up mark, the pooled percent fibroid volume shrinkage difference between UAE (50.57 ± 15.70%) and MRgHIFU (30.06 ± 12.76%) was statistically significant (*p* = 0.0001), with minimum and maximum fibroid volume shrinkages ranging from 32.18 to 78.3% for UAE, and 12.6–50.2% for MRgHIFU.

For 12 months follow-up, three papers reported fibroid volume changes for UAE and another three for MRgHIFU, showing a pooled statistically significant difference (*p* = 0.0001) between UAE (62.78 ± 17.10%) and MRgHIFU (25.91 ± 12.64%). The minimum and maximum percent fibroid volume reduction ranged from 51.7 to 91.2% for UAE and 9.3–38.0% for MRgHIFU. The minimum percent fibroid volume shrinkage at any followup measurement for any treatment was 9.3% for MRgHIFU.

Only one UAE treatment paper, with 98 patients, and two MRgHIFU treatment papers reported percent fibroid volume shrinkage after 24 months, showing a statistically significant difference (*p* = 0.0001) between pooled percent fibroid volume shrinkage between UAE (68.18%) and MRgHIFU (34.96%).

Seven papers reported complications for UAE and twelve papers for MRgHIFU. The most common reported complications were fever, pain, nausea, vomiting, anorexia, fatigue, abdominal distension, transient and permanent amenorrhea (Table 4).

**Table 4:** Complications following UAE and MRgHIFU treatments

#	Complications	UAE (# of complications/all patients, # of studies)	MRgHIFU (# of complications/all patients, %)	*p* value
1	Fever	19/736, 1 study	0/689	< 0.0001
2	Pain	123/736, 4 studies	168/689, 7 studies	0.0004
3	Transitory sciatic neuralgia	0/736	2/689, 2 studies	0.2336
4	Numbness	0/736	15/689, 2 studies	< 0.0001
5	Nausea, vomiting	56/736, 3 studies	7/689, 2 studies	< 0.0001
6	Anorexia	35/736, 2 studies	0/689	< 0.0001
7	Migraine	1/736, 1 study	0/689	1.0000
8	Fatigue	35/736, 2 studies	0/689	< 0.0001
9	Discharged myoma debris	2/736, 1 study	0/689	0.5002
10	Fibroid expulsion	13/736, 4 studies	0/689	0.0003
11	Oligomenorrhea	3/736,1 study	0/689	0.2503
12	Transient amenorrhea	17/736,4 studies	0/689	< 0.0001
13	Permanent amenorrhea	8/736, 3 studies	1/689, 1 study	0.0394
14	Bladder compression syndrome	3/736, 2 studies	1/689, 1 study	0.6253
15	Upper urine tract infection	1/736, 1 study	1/689,1 study	1.0000
16	Inguinal hematoma	9/736, 3 studies	0/689	0.0040
17	Bilateral pulmonary embolism	1/736, 1 study	0/689	1.0000
18	Abdominal distension	34/736, 2 studies	4/689, 1 study	< 0.0001
19	Skin lesion	0/736	61/689, 8 studies	< 0.0001
20	Pruritic rash related to the procedure	11/736, 2 studies	0/689	< 0.0010
21	infection of the necrotic fibroid	0/736	1/689, 1 study	0.4835

Statistically significantly number of cases of complications of fever (*p* < 0.0001), anorexia (*p* < 0.0001), migraine (*p* = 1.0000), transient amenorrhea (*p* < 0.0001), fibroid expulsion (*p* = 0.0003), inguinal hematoma (*p* = 0.0040), fatigue (*p* < 0.0001) and pruritic rash (*p* < 0.0010) were only reported for UAE treatment, with no cases reported for MRgHIFU. Statistically significant numbers of cases of complications only reported for MRgHIFU included numbness (*p* < 0.0001) and skin lesions (*p* < 0.0001, with skin lesions defined as skin redness, edema or superficial skin burns. During management of patients, complications, such as permanent amenorrhea (*p* = 0.0394), and abdominal distention (*p* < 0.0001) were more common with patients treated with UAE. UAE-treated patients treated Patients following MRgHIFU treatment were more likely to report pain than UAE treated patients (*p* = 0.0004).

## Discussion

Our systematic review analyzed and compared fibroid volume shrinkage and common complications following UAE and MRgHIFU procedures, two noninvasive options for the treatment of uterine leiomyoma. Effective fibroid volume shrinkage was reported following both treatments for all reported follow-up periods. Weighted mean percent fibroid reduction was higher for UAE than MRgHIFU in 6–24 months follow-up. Burn et al. 2000 reported the total disappearance of fibroids in one patient 6 months after UAE treatment. The percent fibroid volume shrinkage for UAE (68.18%) at 24 month follow-up was double that of MRgHIFU (34.96 ± 4.88%, *p* = 0.0001). However, Nagiub et al. [16] reported that 7 patients showed percent fibroid volume increase of 8.3% after one year of UAE treatment. Fibroid shrinkage after MRgHIFU was found to be higher than UAE after 3 months follow-up, but was not statistically significant (*p* = 0.068).

In addition, we found that for UAE the percent of fibroid volume shrinkage increased with follow-up time, almost doubling from 3 months follow-up (35.59%) to 24 months (68.18%). This may be explained by the gradual effect of UAE treatment, associated with progressive ischemia of the leiomyoma due to blockage of the uterine artery that supplies it. However, there is also a risk of fibroid regrowth due to collateral blood supply with ovarian arteries [38]. For MRgHIFU percent fibroid volume shrinkage was also substantial, between 25 and 40%, possibly explained by local targeting fibroid tissue with thermal ablation, consequently leading to cell death and fibroid shrinkage [39]. Further studies are needed to identify and compare the progression of fibroid shrinkage following UAE and MRgHIFU treatments.

We found that the numbers of patients complaining reporting nausea and vomiting, permanent amenorrhea and abdominal distension were greater for UAE than MRgHIFU. Whereas, the number of patients reporting pain was larger for MRgHIFU than UAE. Toor et al. 2012 reported that common complications for UAE include deep vein thrombosis, pulmonary embolism and permanent amenorrhea [40]. In our systematic review, we only found one reported UEA-treated case with bilateral pulmonary embolism (1 case/736 UEA treated patients) and none for MRgHIFU (0 cases/689 MRgHIFU treated patients). There were no reported cases with deep vein thrombosis. Commonly reported MRgHIFU complications found in our systematic review our such as skin lesion, numbness, were rare or non-existent among complications reported by UEA patients Sciatic neuralgia, and infections of the necrotic fibroid were rare or absent in our review for either MRgHIFU or UAE treated patients [41]. UAE patients reported more of the following complications than MRgHIFU patients: fever, nausea/vomiting, anorexia, fatigue, fibroid expulsion, transient and permanent amenorrhea, inguinal hematoma, abdominal distension, and pruritic rash; MRgHIFU reported more pain, numbness and skin lesions.

This is the first systematic review comparing with statistical testing the uterine fibroid volume shrinkage and procedural complications for the uterine leiomyoma noninvasive treatments UAE and MRgHIFU. Previously, Taheri et al. [7] conducted a systematic review analyzing papers with more than 20 patients with symptomatic leiomyomas after UAE, radiofrequency ablation, or ultrasound guided HIFU. They stratified the result on different follow-up times after 3, 6, 9, 12 and 26 months. This review found for fibroid shrinkage for UAE was found greater than UgHIFU after all follow-up times, but did not determine if differences found were statistically significant and did not exclude patients with prior treatments, other pelvic diseases or postmenopausal patients (which can contribute to confounding biases). The effectiveness of UAE might be reduced in postmenopausal women due to lower estrogen levels, given the estrogen-dependent pathogenesis of uterine fibroids [42]. Another previous systematic review and meta-analysis conducted by Liu et al. [43] also reported that having previous myomectomies before UAE treatments potential bias for their systematic review and meta-analysis. This systematic review and meta-analysis compared quality of life, re-intervention rate and incidence of adverse events following UAE and MRgHIFU as treatments for uterine leiomyoma. However, this study did not compare fibroid volume shrinkage between these treatments, and only seven papers were included for this analysis, in contrast to the 29 papers in our systematic review.

Our current systematic review compared, for the first time, fibroid volume shrinkage and complications, utilizing statistical testing, for the two non-invasive UAE and MRgHIFU treatments for uterine leiomyoma. There are several strengths and limitations of this study. To minimize bias, we excluded patients with previous treatments for leiomyoma, other pelvic diseases and postmenopausal women to reduce potential confounding bias. Furthermore, we included a total of 29 papers for both the qualitative and quantitative analyses. Fibroid volume shrinkage results were stratified and analyzed by different follow-up times. However, there are several limitations in this study. First, it included only studies published in English., Second, the study did not assess re-intervention rates, symptomatic improvements, quality of live, pregnancy and ovarian reserves, and cost differences between the two procedures. Third, all types of fibroids, including subserosal, submucosal, intramural and pedunculated were included in our systematic review. Further studies are needed to stratify fibroid leiomyoma nodules based on anatomic location to reduce confounding biases, especially because intramural fibroids may have higher percent fibroid volume shrinkage after UAE [42].

## Conclusion

Comparing the effectiveness and safety of these two noninvasive treatments for uterine leiomyoma are essential for assuring best practices in clinical treatment of this condition. Our systematic review identified significant findings on differences between UAE and MRgHIFU by comparing fibroid volume shrinkages and post-procedural complications between treatments. The pooled weighted percent fibroid volume shrinkage for UAE treatment was statistically significantly greater than MRgHIFU at the 6, 12 and 24 months follow-up times, though both treatments showed substantial shrinkage However, UAE was more strongly statistically associated with procedural complications like fever, nausea and vomiting, anorexia, fatigue, fibroid expulsion, transient and permanent amenorrhea, inguinal hematoma, abdominal distension and pruritic rash than MRgHIFU. These findings should contribute to informing women and their physicians on making the best choice of treatment for their needs. Randomized controlled trials, are needed to further validate these findings.

## Data Availability

All study materials are available pre request sent to the corresponding author via email: gulzanat.aimagambetova@nu.edu.kz.
